# Incomplete Kawasaki disease with muscular weakness and bladder retention: a case report

**DOI:** 10.1186/s12887-024-04874-0

**Published:** 2024-06-26

**Authors:** Yating Sang, Lili Luo, Lina Qiao

**Affiliations:** 1 Pediatric Intensive Care Unit, West China Second University Hospital, Sichuan University, Chengdu, China; 2 Key Laboratory of Birth Defects and Related Diseases of Women and Children, Ministry of Education, Sichuan University, Chengdu, China; 3 NHC Key Laboratory of Chronobiology, Sichuan University, Chengdu, China

**Keywords:** Kawasaki disease, Muscular weakness, Bladder retention, Rectal bladder dysfunction, Intravenous immunoglobulin

## Abstract

**Background:**

Kawasaki disease (KD) is an acute systemic immune vasculitis affecting multiple organs and systems in children, and is prevalent in children under 5 years of age. Muscular weakness is a rare manifestation of KD, and only 11 pediatric patients with KD combined with muscular weakness have been reported, of which evidence of myositis was found in 2/3 of the patients, and 1/3 could not be explained by myositis, the mechanism of which is still unclear. Cases of KD combined with bladder retention are even more rare, and there has been only 1 case report of KD combined with bladder retention in a child with no previous underlying disease.

**Case presentation:**

We report a 22-month-old Asian child with incomplete Kawasaki disease (IKD) who initially presented with fever and progressive muscular weakness in the lower extremities, followed by the bladder and bowel retention abnormalities and rapid onset of heart failure, respiratory failure and shock. The child developed coronary artery ectasia (CAA) without the main clinical features of KD such as rash, conjunctival congestion, desquamation of the extremity endings, orofacial changes and enlarged lymph nodes in the neck. Creatine kinase and electromyography were normal. Temperature gradually normalized and muscle strength recovered slightly after intravenous immunoglobulin. The child could be helped to walk after 1 week of aspirin combined with steroid therapy.

**Conclusions:**

We present the case of a 22-month-old child with IKD. The child began with progressive muscular weakness in the extremities, followed by the bladder and bowel retention abnormalities, and rapidly developed heart failure, respiratory failure, and shock. Despite early failure to detect the disease, the child recovered rapidly and had a favorable prognosis. KD comorbidities with muscular weakness as the main manifestation are uncommon. This is the first case report of IKD combined with both muscular weakness and bladder and bowel retention, which may provide clinicians with diagnostic and therapeutic ideas, as well as a basis for future exploration of the mechanisms of KD combined with muscular weakness or bladder and bowel retention abnormalities.

## Introduction

Kawasaki disease is a systemic mesangial vasculitis disease of unknown etiology, commonly seen in children under 5 years of age [1], with the most important complication being involvement of the coronary arteries and the development of CAA or coronary artery aneurysm (CAE). The diagnosis of KD relies on clinical features, systemic multisystemic vasculitis manifestations, and laboratory tests. The main clinical features include 1) fever; 2) changes in the extremities (reddening, swelling and peeling of the skin) 3) bilateral conjunctivitis; 4) changes of lips and oral cavity: reddening of lips, strawberry tongue, diffuse injection of oral and pharyngeal mucosa; 5) redness of the skin rashes or at the site of BCG inoculation; and 6) nonsuppurative enlargement of the lymph nodes in the neck. Complete Kawasaki disease (CKD) is diagnosed when fever and 4 or more other clinical features are present. Children with fewer than 4 major clinical features may be evaluated for IKD in conjunction with laboratory tests and echocardiography (see guidelines for details  [2, 3]).

Children with KD can be combined with multi-organ and multi-system injuries, including gastrointestinal (vomiting, diarrhea, intestinal obstruction, etc.), neurological injuries (aseptic meningitis, encephalopathy, impaired consciousness, etc.), and urological (aseptic pyuria, urethritis, etc.) [4]. To date, 11 cases of children with KD have been reported with combined muscular weakness, mainly characterized by difficulty in walking, ptosis, ocular dyskinesia, respiratory failure, dysphonia and dysphagia [5–15]. The objective of the study is to report a child diagnosed with IKD combined with Kawasaki disease shock syndrome (KDSS), characterized by muscular weakness of the extremities and the bladder and bowel retention abnormalities, who was effectively treated with gammaglobulin, aspirin, and steroid, which has not been reported previously.

## Case report

A 22-month-old boy presented with fever and weakness of the extremities for 3 days. The initial symptoms were decreased activity and abnormal walking gait. he child was treated with a 3-day anti-infection therapy of cefazolin and ceftizoxime at another hospital. Before using antibiotics, they collected the child’s blood for pathogen culture, and the results were negative. However, the child continued to have recurrent temperatures, and developed symptoms such as inability to walk, unsteadiness in holding objects, and choking on drinking water. More importantly he develpoed the bladder and bowel retention abnormalities, and required catheterization. Abdominal CT suggests enlarged gallbladder (See Table 1 for relevant examinations).

**Table 1:** Timeline of relevant testing results done for the patient

	White blood cell count(× 10^9/L)	neutrophil count(× 10^9/L)	Platelet count(× 10^9/L)	C-reactive protein(mg/L)	Procalcitonin(ng/mL)	ESR(mm/h)	IL-6(pg/ml)	Albumin (g/L)	Alanine transaminase (U/L)
Day1-2 (before admission)	17.15	12.04	163	45.79	6.27	NA	NA	NA	NA
Day3 (6 h within admission)	16.3	11.99	240	119.6	6.75	NA	NA	34.5	176
Day4 (6 h after admission)	18.2	15.76	236	138	NA	39	9.97	29.6	179
Day5-7	12.9 → 11.3	9.38 → 7.21	239 → 285	99 → 29.4	NA	NA	NA	33.4	108
Day8 to 10	17.7	11.43	291	11.1	NA	16	NA	39.4	46
Day11-17	9.1	5.02	445	1.2	NA	NA	NA	NA	NA
3 months later	NA	NA	NA	NA	NA	NA	NA	NA	NA
1 year later	NA	NA	NA	NA	NA	NA	NA	NA	NA

	D-Dimer(mg/L FEU)	Urinary leukocytes	Creatine kinase(U/L)	Myocardial markers troponin(ng/L)	Brain natriuretic peptide(pg/ml)	cerebrospinal fluid	2-Dimensional echocardiography	ECG	Other relevant manifestations and laboratory parameters
Day1-2 (before admission)	NA	NA	NA	NA	NA	NA	NA	NA	NA
Day3 (6 h within admission)	2.12	NA	33	253	NA	Nucleated cell count (20 × 10^6^/L) Protein (151.7 mg/L) No bacterial growth mNGS: negative	NA	junctional escape rhythm, sino-auricularblock?	2019-nCOV qPCR from the nasopharynx: negative(no serologic test); blood, CSF and urine culture: negative; blood mNGS: moderate levels of Haemophilus influenzae and Microbacterium fragilis; CSF mNGS, nucleic acids of echovirus-11/30, Enterovire-71 and Cox-A6/16/10, EBV, Cox-IgM, Mycoplasma pneumoniae-IgM, HSV-IgM, fungi and M.tuberculosis: negative;
Day4 (6 h after admission)	6.38	+ + +	NA	NA	22,286	NA	①left ventricular systolic dysfunction (EF = 49% FS = 24%) → normal (EF = 58% FS = 29%) ②tricuspid regurgitation (moderate-severe) → (mild) No coronary artery diameter measurements	sinus tachycardia, nonspecific ST segment changes (II III aVF V3 V5)	NA
Day5-7	27.31	normal	NA	46	NA	NA		(Day7) 24-h holter: normal	NA
Day8 to 10	3.08	normal	NA	NA	NA	NA	LCA = 2.8 mm (2.34z) LAD = 2.0 mm (1.07z) LCX = 1.5 mm (-0.10z) RCA = 2.1 mm (1.22z) Normal left ventricular function (EF = 69% FS = 37%)		NA
Day11-17	0.42	NA	NA	NA	NA	(Day15) Nucleated cell count (0 × 10^6^/L) Protein (153.7 mg/L)	LCA = 2.8 mm (2.34z) LAD = 1.7 mm (0.19z) LCX = 1.3 mm (-0.60z) RCA = 1.8 mm (0.30z) Normal left ventricular function (EF = 72% FS = 39%)		Cranial and spinal MRI: normal (Day 15) EMG: normal (Day 17)
3 months later	NA	NA	NA	NA	NA	NA	LCA = 2.5 mm (1.52z) LAD = 1.6 mm (-0.14z) LCX = 1.4 mm (1.52z) RCA = 1.9 mm (0.62z) Normal left ventricular function (EF = 71% FS = 39%)		NA
1 year later	NA	NA	NA	NA	NA	NA	LCA = 2.6 mm (1.8z) LAD = 1.8 mm (0.50z) LCX = 1.4 mm (-0.24z) RCA = 2.2 mm (1.51z) Normal left ventricular function (EF = 67% FS = 36%)		NA

Coronary artery Z scores reference: Dallaire F, Dahdah N. New equations and a critical appraisal of coronary artery Z scores in healthy children. J Am Soc Echocardiogr 2011;24:60–74

^*^LCA left coronary artery, *LAD* left anterior descending artery, *LCX* left circumflex artery,* RCA* right coronary artery, *EMG* electromyography, *NA* Not applicable

On admission (3th day of illness), the boy was noted to have muscular weakness with diminished patellar tendon reflex. The proximal and distal muscle strength of upper limbs was scaled as grade IV, and lower limbs grade III-IV (See Table 2 for muscle strength scale). Six hours after admission (Day4), the child developed shallow coma, oliguria, decreased blood pressure (85/36 mmHg), respiratory failure (FiO2 50%, PEEP 5 cmH2O), muffled heart tones, hepatomegaly, and a Glasgow score of 6 (E1M4V1). The child required endotracheal intubation for respiratory support, urinary catheterization and other supportive therapy,including continuous pumping of epinephrine, norepinephrine, and furosemide (Day4-6). At the same time, the child was treated with intravenous immunoglobulin (a total of 2 g/kg, infused on Day4 and Day5 respectively) and dexamethasone (0.32 mg/kg, Day 4–7). Because of persistent anemia, coagulopathy, and hypoalbuminemia, multiple transfusions of red blood cells, plasma, and albumin were administered to correct the condition.

**Table 2:** Muscle strength scale

0	Only a trace of movement is seen or felt or fasciculations are present
1	Only a trace of movement is seen or felt or fasciculations are present
2	Muscle can move only if the resistance of gravity is removed (eg, in the horizontal plane)
3	Muscle strength is further reduced such that the joint can be moved only against gravity with the examiner’sresistance completely removed
4	Muscle strength is reduced but muscle contraction can still move joint against resistance
5	Muscle contracts normally against full resistance
*Reference: Medical Research Council. Aids to the Examination of the Peripheral Nervous System. Memorandum no. 45. London, Her Majesty’s Stationery Office, 1981	

On day 7 of illness, he stopped having fever (the last time of fever was 24 h after stopping the immunoglobulin infusion). Circulation was stable. Self-breathing test was passed and he was discharged from invasive ventilator-assisted ventilation therapy. Glasgow coma score improved to10 (E4M4V1).

**Fig. 1:**
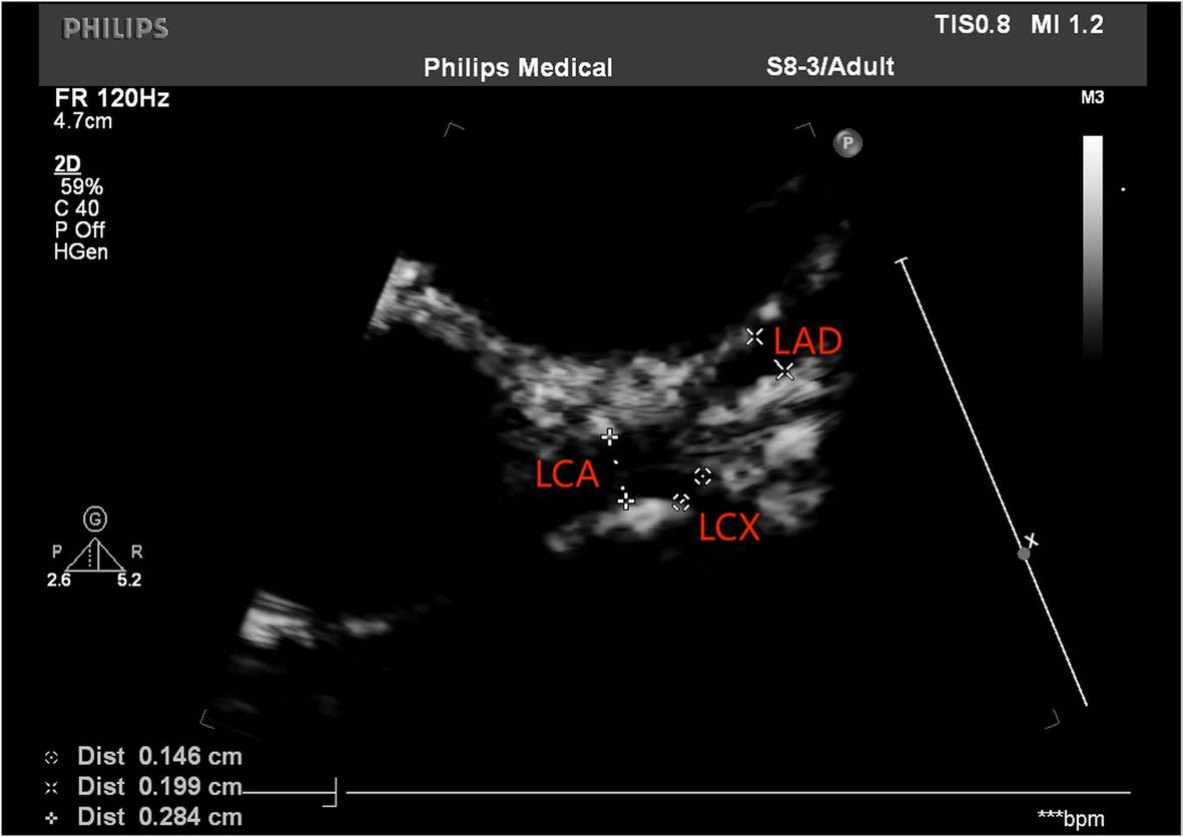
Ultrasound measurement of the left coronary artery and its branches

**Fig. 2:**
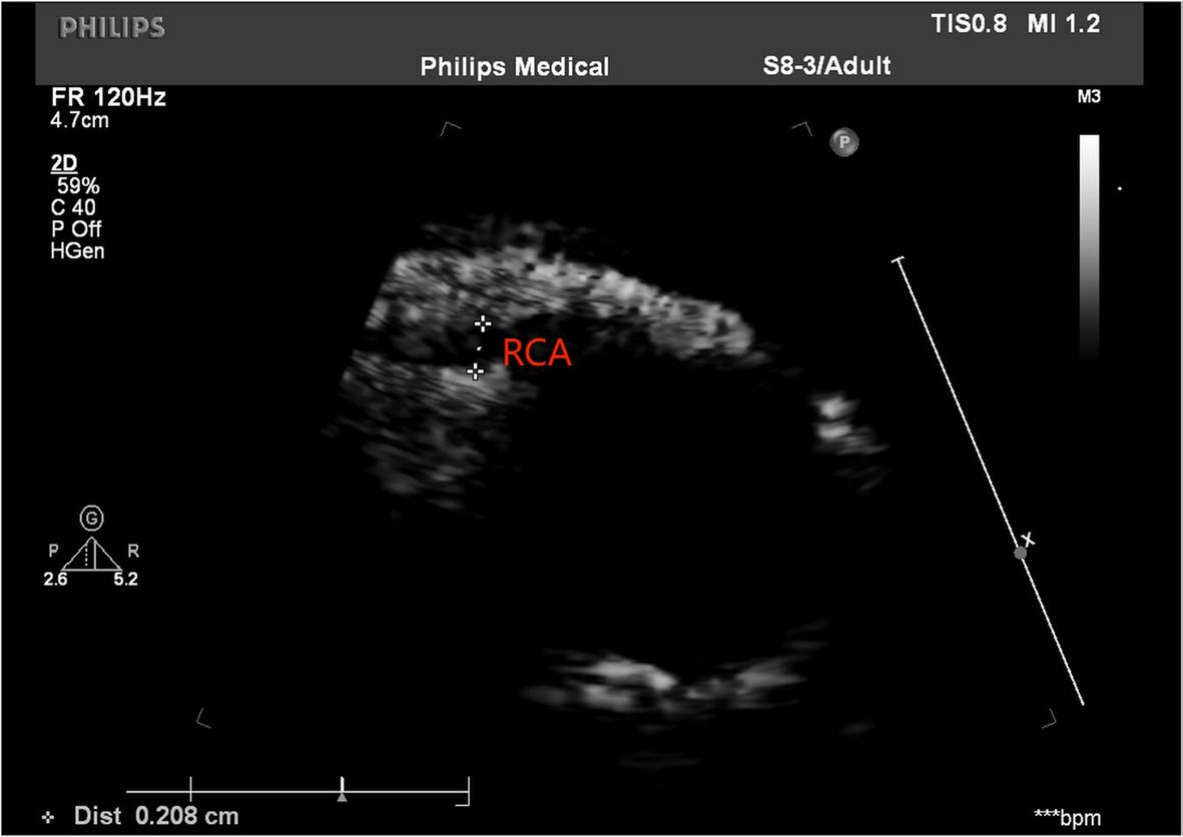
Ultrasound measurement of the right coronary artery

On day 9 of illness, a repeat echocardiogram suggested coronary artery ectasia (LCA = 2.8 mm, LAD = 2.0 mm, LCX = 1.5 mm, RCA = 2.1 mm, *Z* value = 2.34, Figs. 1 and 2). Combined with other tests and the child's manifestations of inadequate tissue perfusion, he was diagnosed with IKD, combined with severe complication of KDSS. We treated with aspirin (4 mg/kg·d) and dexamethasone (0.16 mg/kg, Day8-9) or prednisone acetate (1 mg/kg, since Day 10). The child was able to pass urine and feces voluntarily. Tendon reflexes were normal. Upper and lower extremity muscle strength was grade IV.

On day 16 of illness, the child could walk dozens of steps with support. At this time, the electromyography (EMG) was normal. He continued to take aspirin (for 6 months) and prednisone (gradually tapered off in the outpatient clinic over a 4-week period).

Gradually the child regained complete muscle power with normal activity over the next 3 months. Echocardiography showed normal coronary Z-scores and no valvular regurgitation.

## Discussion and conclusions

This child was unusual in that he did not have the typical clinical manifestations of KD throughout the course of his illness, except for fever, which began with progressive muscular weakness in the extremities, followed by the bladder and bowel retention abnormalities. Therefore, we initially considered Guillain-Barré syndrome, but cerebrospinal fluid examinations performed on days 3 and 15 of the child’s illness showed no protein cell separation. All four central demyelination tests in the cerebrospinal fluid were negative. Cranial and spinal MRI results were normal. EMG was normal. And the child has persistent bladder and bowl dysfunction. But we did not test for GBS-related antibodies. Subsequently, the child rapidly developed multi-organ dysfunction and shock. So we considered infection-related septic shock. However, the child’s blood and cerebrospinal fluid cultures were negative (Specimens were collected prior to the administration of antibiotics at our hospital), and the efficacy of antibiotics (vancomycin and meropenem, 6 days in total) was inconclusive. No rare pathogens were detected in the his blood and cerebrospinal fluid mNGS, such as Rickettsia, Leptospira and so on. We only detected moderate levels of Haemophilus influenzae and Microbacterium fragilis in the blood mNGS. We believe that these two etiological infections do not explain the severe clinical manifestations and systemic inflammatory responses of the child. No link has been found between Kawasaki disease and Haemophilus influenzae or fragile bacteroides. No nucleic acids of echovirus-11/30, Enterovire-71, Cox-A6/16/10 were detected. Tests for EBV, Cox-IgM, Mycoplasma pneumoniae-IgM, HSV-IgM, fungi and M.tuberculosis were negative. Therefore, we did not consider the possibility of infection by rare pathogens. Fortunately, we used high-dose intravenous immunoglobulin (2 g/kg) at the same time, and the child’s temperature gradually normalized within 36 h. C-reactive protein was essentially normalized within 48 h of normalization of temperature. No further dilation of the coronary arteries. No predisposition for macrophage activation syndrome. Therefore there is no indication for the use of biological agents such as infliximab. We retrospectively diagnosed KDSS until the child developed coronary artery ectasia.

According to the American Heart Association (AHA) guidelines [16], Kawasaki disease can be diagnosed when a patient meets the following clinical criteria: 1) Persistent high fever ≥ 5 days with at least 4 main characteristics: rash, bilateral bulbar conjunctival congestion, oral mucosal changes, peripheral limb changes, cervical lymph node enlargement, and other similar clinical features were excluded. When a child has an unexplained fever more than 5 days, incomplete or atypical Kawasaki disease should be considered in conjunction with laboratory and echocardiographic results. The child had no major symptoms other than fever for more than 5 days and coronary dilation. Combined with the clinical manifestations of the child and all auxiliary examinations, other evidences supporting the diagnosis of IKD/KDSS include: 1) Cardiovascular system: left ventricular function transient systolic dysfunction, valve regurgitant, shock; 2) Digestive system: gallbladder enlargement and thickening of gallbladder wall, intestinal obstruction; 3) Respiratory system: interstitial changes in both lungs with a small amount of pleural effusion on both sides; 4) Nervous system: aseptic meningitis (CSF nucleated cells number 20 × 10^6/L, and CSF culture and mNGS were negative); 5) Urinary system: sterile pyuria (urine routine leukocytosis, urine culture negative); 6) Laboratory examinations: neutrophilic leukocytosis, anemia and the trend of thrombocytosis; CRP, ESR increase; hypoalbuminemia and hyponatremia; BNP increased significantly.

CAA has a variety of etiologies in childhood, in addition to the most common Kawasaki disease, other etiologies including juvenile idiopathic arthritis, multiple Arteritis, systemic lupus erythematosus, etc. However, with the exception of KD, None of these other diseases could explain the manifestations of systemic inflammatory responses and multisystem involvement. Since this case occurred in the era of covid, we should also consider the possibility of multi-system inflammatory syndrome in children (MIS-C), which may be caused by a state of excessive inflammation that usually occurs in the weeks following infection. MIS-C is a syndrome of multisystem involvement in the context of severe acute respiratory syndrome coronavirus 2 (SARS-CoV-2) outbreak epidemics, including persistent fever, gastrointestinal symptoms, myocardial injury, shock, and coronary artery aneurysm [17–19]. Nearly half of the patients with MIS-C will present with neurologic manifestations. The muscle involvement may vary from an asymptomatic elevation of CK to severe rhabdomyolysis [20]. However, our child did not present with a rash, no acute kidney injury and myoglobinuria, normal CSF results, negative demyelinating antibodies, and normal spinal MRI and EMG. So causes other than viral myositis can be excluded. Musclular weakness can be the first manifestation of COVID-associated viral myositis, the mechanism of which may be direct infiltration of myocytes by the SARS-CoV-2 virus or induced by autoimmunity [21]. CK may not be elevated, which is consistent with our child. However, our child also showed urinary retention, which has only been reported in patients with MIS-C combined with acute transverse myelitis (ATM) [22, 23]. CSF and spinal MRI were normal in our child. So the diagnosis of ATM was not supported. There was overlap between patients with MIS-C and KDSS compliant clinical presentations and complementary tests. Coronary dilation, myocardial damage, elevated markers of inflammation and cytokines can also be tested in MIS-C. Imaging changes of lung and gastrointestinal symptoms are also common. However, the evidence that does not support MIS-C is as follows: 1) The onset peak of MIS-C is more than 6 years old [24], and the age of children is younger; 2) The common gastrointestinal symptoms of MIS-C include vomiting, abdominal pain and diarrhea, but no cases of intestinal obstruction have been reported until now, which is inconsistent with our children; 3) Patients with MIS-C often have electrocardiogram changes, such as conduction blocks and arrhythmias, but this child’s electrocardiogram showed only sinus tachycardia; 4) Importantly, the child tested negative for SARS-CoV-2 nucleic acid from the nasopharynx. No history of SARS-CoV-2 infection in last 6 weeks, and no SARS-CoV-2 vaccination. From this point of view, the diagnosis of MIS-C is not valid. The suspicious history is that the child presented with nasal congestion and runny nose in the week before the onset of the disease, but there was no fever or cough. The serological test of COVID-19 that our hospital can perform was SARS-CoV-2 antibody, but unfortunately we did not test for this child. However, the serologic test for SARS-CoV-2 has a low positive rate in the days or weeks after infection. The primarily use is to determine prior exposure, and is very limited in the diagnosis of acute infection [25, 26]. Therefore, we continue to analyze the mechanisms of combined muscular weakness and the bladder and bowel retention abnormalities in children, based on the diagnosis of Kawasaki disease, but we must consider the possibility of an atypical MIS-C.

In previous case reports of KD combined with muscular weakness (Table 3), evidence of myositis was found in 2/3 of cases ((i) elevated creatine kinase or (ii) EMG or muscle biopsy supportive of myositis). In other 1/3 of cases, the creatine kinase and EMG were normal. They did not seem to be explained by myositis. In our case, no evidence of myositis was found (creatine kinase, myoglobin and EMG were normal) [27]. All relevant examinations revealed no evidence of neuromuscular disease. The child’s muscle strength consent also returned to normal soon after we gave him gammaglobulin, aspirin and prednisone acetate treatment. A 40-year-old adult patient was diagnosed with Kawasaki disease, associated with significant distal motor and sensory neuropathy, electromyographic abnormalities, and elevated creatine kinase levels. The biopsy of a distal muscle showed immunoglobulin deposition in the sarcolemma. Immunologic evaluation showed high concentrations of cryoglobulins and circulating immune complexes. It is hypothesized that the manifestations of combined musculo-neuropathy in KD may be mediated by immune complexes deposition in vessels and tissues [20].

**Table 3:** Review of all reported cases of Kawasaki disease (KD) with myositis

NO	Age / Sex	Kawasaki disease	Musle involvement	CK	EMG/Muscle biopsy	CAA	Treatment	References
1	18 month/M	Atypical	Isolated lower extremity muscle weakness (left > right)	Normal	Myositis	Normal	Aspirin only (100 mg/ kg/day)	[5]
2	8 year/M	Complete	Diffuse weakness of all extremities (proximal > distal) and Respiratory failure	2371	Myositis Type IIB atrophy and focal muscle fiber degeneration without evidence of vasculitis or inflammatory infiltrates	Coronary aneurysms	Aspirin (100 mg/ kg/day) IVIg (400 mg/ kg/day) Prednisone	[6]
3	3 year/M	Complete	Diffuse weakness of all Extremities (proximal > distal)	152	Myopathic change with fibrillation potentials at rest	Coronary aneurysms	Aspirin only (50 mg/kg)	[7]
4	10 year/F	Complete	Weakness of hip exten- sors and difficulty in getting up from squatting position	Normal	NA	Normal	IVIg Aspirin Prednisolone (2 g/kg/d)	[8]
5	3 year/F	Atypical	Lower limb hypomobility and ptosis	NA	Myositis of the right quadriceps muscle, multiple inflammatory lesions in the upper extremities	Normal	IVIg only (2 g/kg)	[9]
6	18 month/F	Complete	Proximal muscle weakness, dysphonia and dysphagia	72	Myositis	NA	Aspirin only (100 mg/kg)	[10]
7	3 year/M	Complete	Diffuse weakness of all Extremities and ptosis	62	Normal	Normal	IVIg Aspirin Methylprednisolone	[11]
8	10 year/M	Atypical	Symmetrical weakness of the proximal muscles of the upper and lower extremities and weakness of the cervical flexors	844	NA	Normal	IVIg (2 g/kg) + Methylprednisolone (30 mg/kg/d) after muscle strength recovery: Aspirin (4 mg/kg/d) + Prednisolone 2 mg/kg/d)	[12]
9	6 year/F	Atypical	Edema over the feet bilaterally as well as swollen left calf	25-76U/L	Muscle biopsy of the left lower leg: mild chronic inflammatory changes	Normal	IVIg + Aspirin + Hydrocortisone	[13]
10	7 year/NA	Atypical	Left iliopsoas myositis	NA	NA	Dilation of both coronary arteries → persistent coronary aneurysms	IVIg + Aspirin + Methylprednisolone(2 mg/kg/d) + infliximab (6 mg/kg/d)	[10]
11	8 month/M	Complete	Impaired ocular motility; deficit of upward gaze in the left eye	NA	Histologic section of the orbicularis oculi muscle: arteritis and myositis foci	Coronary aneurysms	IVIg (2 g/kg) + Aspirin (100 mg/kg/d) + Methylprednisolone (30 mg/kg/d)	[14]

^*^CK Creatine kinase, *EMG* Electromyography, *CAA* Coronary artery abnormalities, *NA* Not available

The incidence of KD combined with intestinal pseudoobstruction is 2%-3%. The mechanism is thought to be KD-induced mesenteric artery vasculitis, leading to intestinal ischemia and associated intestinal muscular plexus dysfunction [28]. Only one case of KD combined with bladder retention has been reported previously. Hoon et al. reported a 35-month-old girl diagnosed with refractory KD combined with paralytic bowel obstruction and loose neurogenic bladder. They did not mention creatine kinase and EMG  [29]. In our case, the child presented with similar bladder and fecal retention. We examined creatine kinase and EMG, and the results were normal. But EMG was performed after the child’s muscle strength had recovered, so the possibility of muscular weakness due to myositis could not be completely excluded. His whole spinal MRI was normal, except for the possibility of tumor compression such as neuroblastoma  [30]. We hypothesized that the cause of urinary and fecal retention could be ischemic vasculitis of the arteries supplying the pelvic nerves caused by KD, resulting in a dysfunction of the coordination of the internal and external anal sphincters and the bladder-distractor-sphincter-pelvic floor muscles. However, case reports of KD combined with bladder retention are very rare, the mechanisms need to be further explored.

In summary, when a child with persistent fever presents with muscular weakness, urinary or fecal retention, and rapid and critical progression of disease, clinicians must consider the possibility of IKD in combination with rare neurologic disorders. Gammaglobulin, aspirin and steroid treatment is effective.

## Data Availability

The datasets used and analyzed during the current study are available from the author Yating Sang (sangyating2022@163.com) on reasonable request.

## Abbreviations

KD: Kawasaki disease
EMG: Electromyography
MIS-C: Multi-system inflammatory syndrome in children
ATM: Acute transverse myelitis
